# Cognitive Normal Older Adults with APOE-2 Allele Show a Distinctive Functional Connectivity Pattern in Response to Cerebral Aβ Deposition

**DOI:** 10.3390/ijms241411250

**Published:** 2023-07-08

**Authors:** Sheng-Min Wang, Dong Woo Kang, Yoo Hyun Um, Sunghwan Kim, Regina E. Y. Kim, Donghyeon Kim, Chang Uk Lee, Hyun Kook Lim

**Affiliations:** 1 Department of Psychiatry, Yeouido St. Mary’s Hospital, College of Medicine, The Catholic University of Korea, Seoul 06591, Republic of Korea; 2 Department of Psychiatry, Seoul St. Mary’s Hospital, College of Medicine, The Catholic University of Korea, Seoul 06591, Republic of Korea; 3 Department of Psychiatry, St. Vincent’s Hospital, College of Medicine, The Catholic University of Korea, Seoul 06591, Republic of Korea; 4 Research Institute, Neurophet Inc., Seoul 08380, Republic of Korea

**Keywords:** APOE, beta-amyloid, functional MRI, default mode network, central executive network, graph theory, Alzheimer’s disease

## Abstract

The ε2 allele of apolipoprotein E (ε2) has neuroprotective effects against beta-amyloid (Aβ) pathology in Alzheimer’s disease (AD). However, its impact on the functional connectivity and hub efficiency in cognitively normal older adults (CN) with ε2 is unclear. We investigated the functional connectivity differences in the default mode network (DMN), salience network, and central executive network (CEN) between A-PET-negative (N = 29) and A-PET-positive (N = 15) CNs with ε2/ε2 or ε2/ε3 genotypes. The A-PET-positive CNs exhibited a lower anterior DMN functional connectivity, higher posterior DMN functional connectivity, and increased CEN functional connectivity compared to the A-PET-negative CNs. Cerebral Aβ retention was negatively correlated with anterior DMN functional connectivity and positively correlated with posterior DMN and anterior CEN functional connectivity. A graph theory analysis showed that the A-PET-positive CNs displayed a higher betweenness centrality in the middle frontal gyrus (left) and medial fronto-parietal regions (left). The betweenness centrality in the middle frontal gyrus (left) was positively correlated with Aβ retention. Our findings reveal a reversed anterior–posterior dissociation in the DMN functional connectivity and heightened CEN functional connectivity in A-PET-positive CNs with ε2. Hub efficiencies, measured by betweenness centrality, were increased in the DMN and CEN of the A-PET-positive CNs with ε2. These results suggest unique functional connectivity responses to Aβ pathology in CN individuals with ε2.

## 1. Introduction

Alzheimer’s disease (AD) is a progressive neurodegenerative disorder characterized by memory loss and cognitive decline [1]. The amyloid hypothesis and tau theory of AD initially had a divergent view and separate explanations for the pathological changes associated with AD [2,3]. The amyloid hypothesis proposed that the accumulation of beta-amyloid (Aβ) protein is the primary driver of AD [4], while the tau theory suggested that the abnormal hyperphosphorylation of tau protein, forming neurofibrillary tangles, and associated neuronal injury are the key pathological features [5]. A contemporary disease model of AD, which converged the amyloid and tau theories, suggested that the deposition of Aβ peptide is an upstream event that is associated with downstream synaptic dysfunction, tau deposition, neurodegeneration, and eventual cognitive decline [6].

Synaptic dysfunction evidenced by functional magnetic resonance imaging (fMRI) is known to reflect the earliest pathological change following cerebral Aβ deposition [7]. Studies have suggested that the brain regions particularly vulnerable to early Aβ deposition are the default mode network (DMN) [8]. The brain regions including the posterior cingulate cortex (PCC), precuneus, medial prefrontal, inferior parietal cortex, lateral temporal cortex, and hippocampus are known to constitute the DMN [9]. It is generally acknowledged that the DMN is deactivated during cognitive task performance and activated when individuals are focused on their internal mental-state processes, such as self-referential processing, interoception, autobiographical memory retrieval, or imagining future [10]. Initial resting state fMRI studies have showed that the DMN functional connectivity is lower in cognitively normal older adults (CN) with cerebral Aβ deposition than in those without an Aβ burden [11,12]. Recent studies have elucidated that, in CNs, the anterior DMN shows an excitatory or compensatory increased functional connectivity and the posterior DMN shows a decreased functional connectivity, or so called anterior–posterior dissociation, in response to an Aβ burden [13]. Other intrinsic networks, including the salience network and central executive network (CEN), are also known to be affected with cerebral Aβ deposition in CNs [14,15,16]. Previous results have shown that the functional connectivity of the salience network is not different, and the CEN is lower in CNs with Aβ deposition than in normal controls [14,17]. Others have shown that Aβ-positive CNs have a higher overall salience network connectivity than Aβ-negative CNs [18].

The functional connectivity measures are bivariate and neglect how the ensemble of connections characterize brain function [19]. Graph measures can quantify the topography of the network, so increasing studies have utilized a combination of resting-state fMRIs and graph theory analyses to investigate the topological organization of whole-brain functional networks [19,20]. Another important advantage of graph theory analysis is that it might describe resting-state compensatory response by exhibiting an increased nodal efficiency [21]. This approach allows for a more nuanced exploration of brain network properties and has the potential to understand the compensatory mechanisms or disruptions in AD. Among the various measures provided by graph theory analysis, betweenness centrality is a particularly relevant and informative measure when studying brain networks. Betweenness centrality, defined as the fraction of all the shortest paths in the network that pass through the node, is known to reflect the amount of information traverse to a node, or the nodal efficiency [18]. Therefore, alterations in betweenness centrality can indicate disruptions in the flow of information and potential breakdowns in network communication [22]. Examining changes in the betweenness centrality within brain networks may identify the regions that play a pivotal role in information transmission and processing. Previous studies have shown that the betweenness centrality was lowered, mainly in the DMN, in patients with a trajectory of AD, more so than in normal controls [19,23]. 

The apolipoprotein E (APOE) gene, a major genetic risk modifier for AD, is also an important factor influencing the functional connectivity pattern in the AD continuum [24]. The aberration pattern of this functional connectivity might vary according to different APOE allotypes [25]. Studies have revealed that, in ε4, which is a risk factor of AD, the aberration of the functional connectivity may manifest even before cerebral Aβ deposition becomes detectable [26]. On the other hand, ε2 is a protective factor for AD and is known to have a diverse neuroprotective mechanism preventing the initiation of Aβ pathology. More importantly, ε2 maintains synaptic functions even during AD pathogenesis [27]. 

Despite the above findings, no previous studies have investigated the effects of Aβ deposition to the three important intrinsic networks in CNs with ε2 without ε4 (ε2/ε2 or ε2/ε3). Rather, most research until now has centered on investigating the functional connectivity difference among ε2, ε3, and ε4. For example, a study showed that the functional connectivity was lower in the right precuneus in CNs with ε2 than in those with ε3/ε3 and ε3/ε4 [28]. Another study showed that the DMN functional connectivity was reduced in the bilateral precuneus and anterior cingulate cortex (ACC) regions for ε2 carriers compared to ε3 homozygotes in CNs [29]. However, none of these studies included patients with Aβ deposition confirmed using an amyloid position emission tomography (A-PET) scan. Thus, whether the efficiency of the functional connectivity hubs is altered in response to Aβ or to other factors is also not understood. The dearth of such research could be contributed to a lower rate of ε2. The global prevalence of the ε2, ε3, and ε4 alleles is estimated as 7, 79, and 14%, respectively, and cerebral Aβ accumulates at a much lower rate in CNs with ε2 than those with ε3 or ε4 [30]. Thus, gathering A-PET-positive CNs with ε2/ε2 or ε2/ε3 might have been very difficult and resulted in a lack of studies in clinical settings. 

We aimed to investigate the functional connectivity differences in the three ICNs (DMN, salience network, and CEN) between A-PET-negative CNs and A-PET-positive CNs with ε2/ε2 or ε2/ε3. We also explored the association between cerebral Aβ and the functional connectivity pattern of these intrinsic networks. In addition, we elucidated whether the functional connectivity efficiency differed according to the cerebral Aβ deposition using graph theory measures.

## 2. Results

### 2.1. Baseline Demographic and Clinical Data

The baseline demographic data of the A-PET-negative (N = 29) and A-PET-positive (N = 15) groups are presented Table 1. All variables were normally distributed and there were no significant differences in age, education, sex, rate of ε2/ε2, and neuropsychological profiles based on the Korean version of the Consortium to Establish a Registry for Alzheimer’s Disease (CERAD-K). The A-PET-positive group had a significantly higher cerebral Aβ deposition and mean global standardized uptake value ratios (SUVR) values than the A-PET-negative group.

### 2.2. Group Difference in Intrinsic Functional Connectivity 

**Figure 1:**
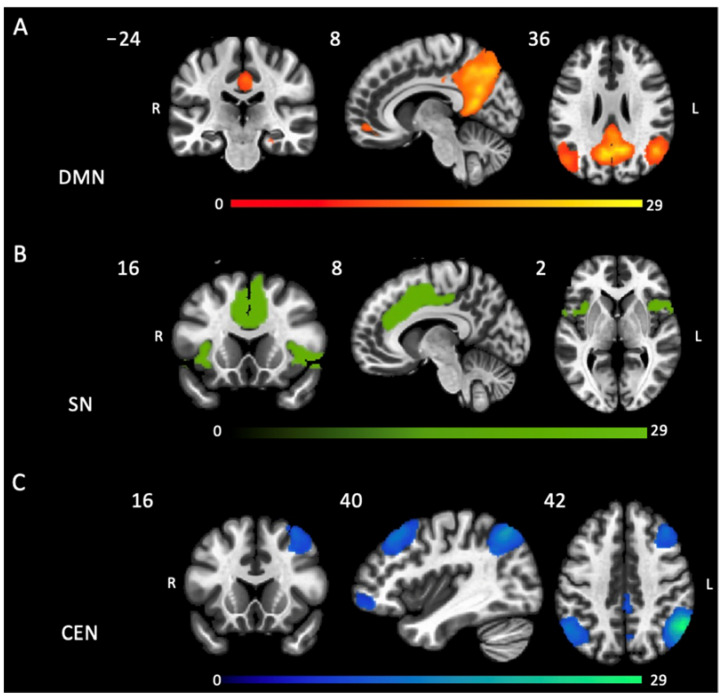
Spatial maps of the resting state intrinsic connectivity networks identified by see-to-voxel analysis of all subjects. (**A**) Default mode network (posterior cingulate cortex as the seed); (**B**) Salience network (anterior cingulate cortex as the seed); and (**C**) Central executive network (right posterior parietal cortex as the seed), for all false discovery rate corrected *p* < 0.001.

A statistical map representing the DMN, salience network, and CEN based on a seed-to-voxel analysis determined across all subjects is shown in Figure 1. 

**Figure 2:**
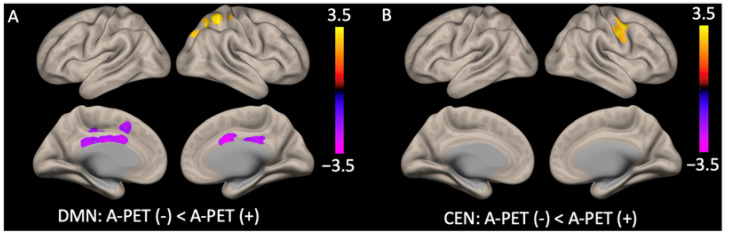
Statistical map representing group difference in default mode network and central executive network determined across all subjects. For (**A**) default mode network, A-PET-positive group showed higher posterior functional connectivity (right superior parietal cortex and precuneus) and lower anterior functional connectivity (anterior cingulate cortex and middle cingulate cortex) than the A-PET-negative group; (**B**) central executive network, A-PET-positive group showed higher functional connectivity in right middle frontal gyrus than the A-PET-negative group (for all *p* < 0.05 false discovery rate corrected).

In terms of the DMN, the group seed-to-voxel analysis, with the posterior cingulate cortex (PCC) as the seed, showed a significantly higher posterior DMN functional connectivity (right superior parietal cortex and precuneus) and lower anterior DMN functional connectivity (anterior cingulate cortex (ACC) and middle cingulate cortex) in the A-PET-positive group compared to the A-PET-negative group (*p* < 0.05, FDR corrected). In the salience network, with the ACC as the seed, there were no significant differences between the two groups. For the CEN, with the right PPC as the seed, the functional connectivity within in the right middle frontal gyrus was higher in the A-PET-positive group than in the A-PET-negative group (*p* < 0.05, FDR corrected) (Figure 2 and Table 2). 

### 2.3. Cerebral Aβ Deposition and Functional Connectivity

**Figure 3:**
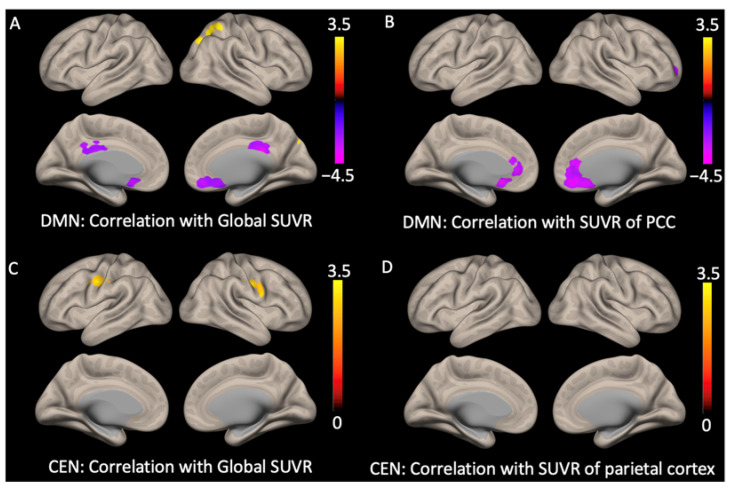
Correlation analysis between functional connectivity and beta-amyloid retention. For default mode network, (**A**) the global mean SUVR scores showed negative correlation with the subgenual anterior cingulate cortex and the posterior cingulate cortex and positive correlation with the precuneus/superior parietal lobule (right); and (**B**) regional mean SUVR scores of posterior cingulate cortex showed negative correlation with the subgenual anterior cingulate cortex. For central executive network, (**C**) the global mean SUVR scores showed positive correlation with the precentral gyrus/middle frontal gyrus; and (**D**) the regional mean SUVR scores showed no correlation (for all *p* < 0.05 false discovery rate corrected).

Figure 3 shows the correlation analysis results between the functional connectivity and cerebral Aβ deposition within the DMN and CEN in all subjects. In the DMN, the global SUVR showed a negative correlation with the anterior region (subgenual ACC) and medial part of the posterior region (PCC). There was also a positive correlation between the global SUVR and the functional connectivity of the posterior region (right superior parietal cortex and precuneus) (Figure 3A and Table 2). We also conducted a correlation analysis between the functional connectivity and regional SUVR of the PCC, which was the seed for our DMN. The regional SUVR of the PCC showed a negative correlation with the anterior DMN (sub-genual ACC) (Figure 3B and Table 2). In the CEN, the global mean SUVR showed a positive correlation with the precentral gyrus and middle frontal gyrus, but there was no significant correlation between the regional mean SUVR and the functional connectivity of the parietal cortex (Figure 3C,D and Table 2) (for all *p* < 0.05 with FDR corrected). 

### 2.4. Graph Theory Measures 

**Figure 4:**
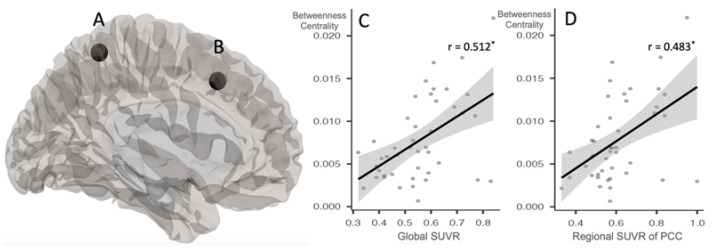
Group difference in graph theory measures and correlation with Beta-amyloid retention. * *p* < 0.05. Betweenness centrality was significantly higher in (**A**) medial fronto-parietal regions (left) and (**B**) middle frontal gyrus (left) in A-PET-positive group than in A-PET-negative group (for both *p* < 0.05 false discovery rate corrected). The betweenness centrality values for middle frontal gyrus (left) showed a positive correlation with (**C**) global SUVR scores and (**D**) regional SUVR scores of posterior cingulate cortex.

The analysis showed that the betweenness centrality was significantly higher in the middle frontal gyrus (L) and medial fronto-parietal regions (L) in the A-PET-positive group than that in the A-PET-negative group (FDR corrected *p* < 0.05) (Figure 4A,B and Table 2). No statistically significant correlations were found between the betweenness centrality values for the medial fronto-parietal regions with those of the global and regional SUVRs. However, the betweenness centrality values for the middle frontal gyrus (L) showed a positive correlation with the global SUVR and regional SUVR of the PCC (Figure 4C,D and Table 2).

## 3. Discussion

Multiple recent studies have sought to investigate the protective mechanism of ε2 in the trajectory of AD. A study found that the fractional amplitude of the low-frequency fluctuation of the inferior parietal lobule was increased in patients with mild cognitive impairment with ε2/ε3 compared to those with ε3/ε3 [31]. Another recent study using data from the Alzheimer’s Disease Neuroimaging Initiative found that CNs with ε2/ε3 had a lower insula functional connectivity compared to CNs with ε3/ε3, while those with mild cognitive impairment with ε2/ε3 had a higher functional connectivity than those with ε3/ε3 [32]. However, the first study only included 10 patients with mild cognitive impairment with ε2/ε3, in contrast with 61 patients with ε3/ε3. In addition, both studies included subjects with diverse degrees of cerebral Aβ deposition, but did not investigate the effect of Aβ in the functional differences between ε2 and ε3. Thus, they were unable to determine whether the functional connectivity differences were attributed to ε2, Aβ, or a combined effect. 

To the best of our knowledge, this is the first study to investigate the effect of Aβ burden on the three large-scale intrinsic networks (the DMN, salience network, and CEN) and the efficiency of their hubs using a graph theory analysis in CNs with ε2/ε2 or ε2/ε3. We found that the functional connectivity of (i) the anterior DMN (antero-middle cingulate) was lower and that of the posterior DMN was higher; (ii) the salience network was not different; and (iii) the CEN was higher in the A-PET-positive group than the A-PET-negative group. In terms of the DMN, it is generally known that CNs exhibit increased anterior and decreased posterior functional connectivity in response to Aβ deposition [33]. We previously showed that this anterior–posterior dissociation has a detrimental effect. Decreased posterior functional connectivity has been associated with episodic memory decrement [34]. In contrast, increased anterior functional connectivity has been associated with depressive symptom severity and an excessive engagement in self-referential introspective thoughts that revolve around past experiences, problems, or distressing events, or so-called rumination [34]. Another study showed that the functional connectivity of both the anterior and posterior DMN was lowered in CNs with the ε2 allele than in CNs with the ε3 allele, which suggested that CNs with the ε2 allele have a distinct functional connectivity pattern [29]. We extended this previous research by showing novel findings of a reversed dissociation pattern of the DMC’s functional connectivity in response to the cerebral Aβ burden. 

ε2 is associated with a slower spreading of Aβ and a more efficient compensatory mechanism against Aβ pathology than ε3 or ε4 [35]. The functional connectivity of the posterior DMN could have been increased in the A-PET-positive group to compensate for the subtle cognitive decline from Aβ deposition [36]. The cingulate gyrus wraps around the corpus callosum like a “belt”, so it can structurally and functionally connect anterior and posterior parts of the brain [37,38]. Since higher neural activity may enhance the production and spreading of Aβ [39], the lower functional connectivity of the antero-middle cingulate might be a protective response against Aβ spreading to various parts of the brain. From another perspective, the Aβ burden is known to be associated with a decrement in synaptic plasticity [7], and the functional connectivity aberrance could have been more predominant in areas known to be more vulnerable to early Aβ deposition, which is a cingulate area [40]. In order to compensate for the lowered functional connectivity of the cingulate regions and subtle cognitive decline from Aβ deposition [36], the functional connectivity of the posterior DMN could have been heightened in the A-PET-positive group. The functional connectivity of the CEN was also reversed, which was higher rather than lower in the A-PET-positive group compared to the A-PET-negative group. A study suggested that the greater frontal lobe functional connectivity in response to Aβ, especially in the CEN, might be due to a compensatory mechanism associated with DMN impairments [15]. ε2 has a greater neurotrophic effect and maintains neuronal survival and synaptic functions under Aβ burden [27,41]. Thus, such a compensatory reaction to Aβ pathology could be more pronounced in CNs with ε2. However, further replication studies with larger sample sizes are needed to clarify this point.

The global Aβ SUVRs had (i) a negative correlation with the functional connectivity of the anterior (sub-genual ACC) and medial parts of the posterior DMN (PCC) and (ii) a positive correlation with the functional connectivity of the posterior DMN (right superior parietal cortex and precuneus). We also observed a negative correlation between the regional SUVR of the PCC with the functional connectivity of the anterior DMN (subgenual ACC). In line with the reversed dissociation pattern of the DMN, our findings suggest that the association between Aβ retention and the DMN functional connectivity are also reversed in ε2, which is in contradiction with the previous study with subjects with diverse APOE alleles [14,42]. Interestingly, the most profound area showing a decreased functional connectivity in association with heightened (global and regional) Aβ was the subgenual ACC, which belongs to the brain regions constituting pathological Braak stage A, one of the earliest brain regions that shows Aβ deposition [43]. We previously showed an “acceleration hypothesis” in response to Aβ [14] in CNs, which suggested that, once Aβ deposition is initiated, a milieu of higher functional connectivity facilitates the deposition and spreading of Aβ, culminating in a decrement of functional connectivity [44]. Thus, the functional connectivity decrement in the cingulate could be a protective response against Aβ spreading to more advanced Braak regions [45]. In addition, an enhanced posterior DMN functional connectivity might result in improved cognition, whereas a decreased anterior DMN functional connectivity might prevent unwanted negative emotional byproducts such as rumination and depressive symptoms [34]. We also observed that the global mean SUVR scores showed a positive correlation with the precentral gyrus and middle frontal gyrus of the CEN. The CEN is strongly associated with working memory and decision making in the context of goal-directed behavior [46,47]. Studies have shown that the CEN and DMN have an anticorrelated functional connectivity, and the anticorrelation of the CEN might be a compensatory response to DMN impairments [14,46]. Taken together, increased CEN functional connectivity could be a compensatory response to lowered anterior DMN functional connectivity, which might result in conserved working memory against Aβ pathology. However, further longitudinal studies are needed to confirm our hypothesis.

The APOE protein is synthesized by astrocytes rather than neurons in the brain, and it interacts with microglia to regulate their response to inflammatory stimuli [48]. Microglia can become activated in response to Aβ deposition and lead to the release of pro-inflammatory molecules and neurotoxic factors, which, in turn, can influence synaptic function and neuronal connectivity. Studies have shown that ε2 is more associated with the anti-inflammatory cascade, whereas ε4 is more closely linked with the pro-inflammatory process when compared to ε3 [49]. En masse, ε2 might exert a protective mechanism against Aβ-associated neurotoxicity by utilizing a more efficient compensatory mechanism, the reversed anterior–posterior dissociation pattern in the DMN and heightened functional connectivity in the CEN. However, subsequent studies using neuroinflammation brain imaging techniques are needed to confirm our speculations. 

In contrast with previous findings [19,23], we observed an increased betweenness centrality for one of the hubs in the CEN (middle frontal gyrus) and DMN (medial fronto-parietal regions). The betweenness centrality of the CEN was also increased in correlation with the global and regional Aβ burden. Since betweenness centrality is known to reflect the quantity of information traverse, our results might indicate that the functional connectivity of each hub region for the DMN and CEN became more efficient in response to Aβ. Interestingly, the regions showing a higher betweenness centrality corresponded to the brain regions showing an increased functional connectivity in response to Aβ. Taken together, the increased posterior DMN functional connectivity and the CEN in the A-PET-positive group might have be related, as a compensatory response to Aβ, with higher efficiencies of these intrinsic networks regions.

Our study has several limitations. This was a cross-sectional study, so we could only report correlations and had a limited ability to infer causal pathways. Further longitudinal analyses are needed to clarify the causal relation among cerebral Aβ, the functional connectivity of the large-scale ICNs, and the efficiencies of this functional connectivity in the trajectory of AD. Second, we only investigated subjects with ε2/ε2 or ε2/ε3. Future studies comparing the functional connectivity change in response to Aβ including diverse alleles (i.e., ε2/ε2 or ε2/ε3 vs. ε3/ε3 vs. ε3/ε4 or ε4/ε4) are needed to elucidate the distinct neurobiological mechanism of each allele. Third, we only had one subject with ε2/ε2, so were not able to explore whether the functional connectivity changes differed between individuals with the ε2/ε2 and ε2/ε3 genotypes, and if so, whether these changes were related to the level of Aβ. This analysis could provide further insights into the interaction between APOE genotypes and Aβ burden. Lastly, the small sample size is another shortcoming. However, a meta-analysis showed that the ages at which 15% of the participants with CN showing A-PET positivity were approximately 40 years for ε4/ε4 carriers, 65 years for ε3/ε3 carriers, and 95 years for ε2/ε3 carriers [30]. Thus, the fact that we were able to acquire age-matched A-PET-positive CN patients could be our strength. 

## 4. Materials and Methods

### 4.1. Subjects

A total of 44 CNs with either the ε2/ε2 or ε2/ε3 allotype, 29 with amyloid PET-negative results (A-PET-negative group), and 15 with amyloid PET-positive results (A-PET-positive group) were included in the study. The subjects were recruited from volunteers in the Catholic Aging Brain Imaging (CABI) database, which contains brain scans of patients who visited the outpatient clinic at Catholic Brain Health Center, Yeouido St. Mary’s Hospital, The Catholic University of Korea, between 2017 and 2022. All the subjects included were aged ≥ 50 years and had normal cognitive function confirmed with the CERAD-K, which includes Verbal Fluency (VF), the 15-item Boston Naming Test (BNT), the Korean version of the MMSE, Word List Memory (WLM), Word List Recall (WLR), Word List Recognition (WLRc), Constructional Praxis (CP), and Constructional Recall (CR) [50]. 

The subjects in the A-PET-positive group were matched to the A-PET-negative group according to age, handedness, education level, sex, and neurocognitive measures. Subjects with any current or past diagnosis of mild cognitive impairment or dementia established by the National Institute on Aging and Alzheimer’s Association criteria were excluded. The diagnosis of normal cognitive status was conducted separately by two psychiatric specialists, and they also confirmed the inclusion and exclusion criteria. The study was conducted in accordance with the ethical and safety guidelines set forth by the Institutional Review Board of Yeouido St. Mary’s Hospital, College of Medicine, The Catholic University of Korea (IRB number: SC22RIDI0153).

### 4.2. Acquisition of MRI

All MRI data were collected by the Department of Radiology, Yeouido St Mary’s Hospital, College of Medicine, The Catholic University of Korea, using a 3T Siemens MAGETOM Skyra machine and 32-channel Siemens head coils (Siemens Medical Solutions, Erlangen, Germany). We used following parameters; (1) the T1-weighted, three-dimensional, magnetization-prepared rapid gradient-echo (3D-MPRAGE) sequence was TE = 2.6 ms, TR = 1940 ms, inversion time = 979 ms, FOV = 230 mm, matrix = 256 × 256, and voxel size = 1.0 × 1.0 × 1.0 mm^3^; (2) the T2-weighted MRI sequences were TE = 91 ms, TR = 3700 ms, flip angle (FA) = 150°, FOV = 220 × 220 mm, matrix = 448 × 448 in-plane resolution, and 3 mm slice thickness. Resting-state fMRIs were collected using a T2-weighted gradient echo sequence with TR = 2000 ms, TE = 30 ms, matrix = 128 × 128 × 29, and voxel size = 1 × 1 × 2 mm^3^. A total of 150 volumes were acquired over 5 min while the patients were instructed to keep their eyes closed and think of nothing in particular.

### 4.3. [^18^F]-Flutemetamol PET Image Acquisition and Processing

Information regarding the production, data collection, and analytic procedures for [^18^F]-flutemetamol (^18^F-FMM) and ^18^F-FMM PET was described previously [51]. Static PET scans were acquired from 90 to 110 min after injecting 185 MBq of ^18^F-FMM. We used T1 MRI images of each individual to co-register, define the regions of interest (ROIs), and correct the partial volume effects associated with the expansion of the cerebrospinal spaces due to cerebral atrophy using a geometric transfer matrix. The standardized uptake value ratios (SUVRs) were used to quantify the ^18^F-FMM uptake on the PET scan. To define the global cerebral Aβ burden, the SUVRs of the six cortical ROIs (frontal, superior parietal, lateral temporal, striatum, anterior cingulate cortex, and posterior cingulate cortex/precuneus) were averaged, with the pons as the reference region. Consistent with the cutoff values used in previous 18F-FMM PET studies, we used a neocortical SUVR of 0.62 as the cutoff between high and low [51], but amyloid positivity was confirmed by visual readings from two separate nuclear medicine radiologists.

### 4.4. Data Analysis

#### 4.4.1. fMRI Data Preprocessing

We used the functional connectivity (CONN) toolbox in Statistical Parametric Mapping 12 to carryout the fMRI data preprocessing [52]. The default CONN preprocessing pipeline was utilized, which included realignment, unwarping, slice-timing correction, co-registering echo planar images (EPIs) to a T1 structural image, normalization, functional outlier detection and scrubbing, functional spatial smoothing with an 8 mm Gaussian kernel, and anatomical component-based noise correction or denoising. The waveform of each brain voxel was filtered using a bandpass filter (0.009 < f < 0.08 Hz) to reduce the effects of low-frequency drift when removing white matter, CSF noise components, unwanted subject motion, and physiological noises.

#### 4.4.2. Seed-to-Voxel Analysis

We used the default seeds of the CONN toolbox, which included a total of 164 regions of interest (ROIs) that can be utilized as seeds. The posterior cingulate cortex (PCC), the ACC, and the right posterior parietal cortex (PPC) are known to be some of the major hubs of the DMN, the salience network, and the CEN, respectively [53,54]. Thus, we selected the PCC, the ACC, and the right PPC as our seeds. The first-level analyses involved a computation of the seed-to-voxel connectivity maps implemented to each subject, which were adopted in the group level-analysis. Thereafter, we utilized between-group difference controlling for sex, age, and education to assess whether there were statistically significant differences between the DMN, the salience network, and the CEN functional connectivity between the A-PET-negative and the A-PET-positive groups. We also conducted a correlation analysis between the functional connectivity measures with the PET SUVRs. All the comparisons throughout the whole brain adopted voxel-wise statistics, which were thresholded at *p* < 0.05 and false discovery rate (FDR) corrected to resolve the problem of multiple comparisons.

#### 4.4.3. Graph Theory Analysis

Graph theory is a standard framework for the mathematical representation of a network, which can be represented as a graph by G (N, K), with N indicating the number of nodes and K as the number of edges in the graph G [21]. Centrality is a simple measurement of the connectivity between a single node and all the other nodes in a network, representing the importance of a node in a network [55]. Among the diverse centralities, betweenness centrality is acknowledged to reflect the efficiency of a hub [56]. The mathematical details related to the computation of betweenness centrality have been described in previous studies [19,21]. Simply, betweenness centrality is defined as the fraction of all the shortest paths in a network that pass through a node, which is associated with the amount of information traversing the node or any given brain region [18]. 

We used the graph theory technique to investigate the topological features of the functional connectivity graphs across multiple regions of the brain [57]. The CONN toolbox enables a computation of both the global and nodal graph measures on binary and weighted networks. Brain regions can be represented as graph nodes, whereas interregional resting-state functional connectivity can be represented as edges. At the first-level analysis, we performed a graph adjacency matrix by computing an ROI-to-ROI analysis for each subject. To extract the best indices of the network organization, te network edges were adjusted with a threshold for a cost higher than 0.15 on a two-sided test for the adjacency matrix. Thereafter, the adjacency matrix was employed for estimating the common features of the betweenness centrality. The between-group differences in the betweenness centrality were determined using two-tailed t-tests with a *p* < 0.05 (FDR-corrected). After the group-level comparisons, the betweenness centrality measure of the ROIs with significantly higher betweenness centralities was extracted for a further correlation analysis with the PET SUVRs.

### 4.5. Statistical Analysis

We used Jamovi (Version 2.3.18.0) to perform the statistical analyses of the demographic and clinical data [58]. A two-sample independent *t*-test and Chi-square test were utilized to assess the potential differences between the two groups (A-PET-negative group vs. A-PET-positive group) for the continuous variables and categorical variables, respectively. A Pearson correlation analysis was utilized to investigate the association between the two continuous variables. In all the analyses, a two-tailed α level of 0.05 was chosen to indicate statistical significance.

## 5. Conclusions

We showed novel findings of a reversed anterior–posterior dissociation pattern in the DMN functional connectivity and heightened CEN functional connectivity in A-PET-positive CNs with ε2. We also advanced previous research by showing that the hub efficiencies for the DMN and CEN were increased in the A-PET-positive CNs with ε2. These findings suggest that CNs with ε2 might exhibit distinct or more efficient compensatory functional connectivity reactions in response to Aβ pathology. 

Emerging research has suggested that tau pathology begins in a much earlier phase of AD than previously believed [59]. With significant advancements in the field of blood-based biomarkers and neuroimaging techniques, plasma phosphorylated tau 181 levels have been shown to predict both the cerebral tau pathology of the tau-PET and the Aβ deposition of the A-PET [60]. Thus, future studies should focus on investigating the interplay among tau pathology, Aβ deposition, APOE, and the functional connectivity in the trajectory of AD. From another perspective, genome editing tool clustered regularly interspaced short palindromic repeats (CRISPR)-Cas9 has emerged as a potential technology for correcting ε4 to ε2 or ε3 [61]. Thus, longitudinal studies containing diverse APOE profiles with larger sample sizes are needed to confirm and expand upon the protective and detrimental mechanism association with APOE in the progression of AD. These investigations, combined with advancements in neuroimaging techniques, network analyses, and therapeutic approaches, hold promise for improving early detection, monitoring disease progression, and developing effective interventions for individuals at risk of or diagnosed with AD.

## Figures and Tables

**Table 1:** Demographic and clinical characteristics of the study participants.

	Amyloid-PET Negative Group (N = 29)	Amyloid-PET Positive Group (N = 15)	*p*-Value
Age (years ± SD)	71.62 ± 8.13	71.67 ± 8.76	NS
Education (years ± SD)	11.69 ± 4.54	11.00 ± 6.43	NS
Sex (M:F)	9:20	4:11	NS
CDR (SD)	0	0	NS
SUVR (mean ± SD)	0.484 ± 0.080	0.695 ± 0.084	<0.01
APOE 2/2:2/3 (APOE 2/2%)	1:28 (3.4%)	0:14 (0%)	NS
CERAD-K Battery (SD)			
VF	14.97 ± 4.41	13.67 ± 5.19	NS
BNT	12.10 ± 2.24	11.50 ± 1.88	NS
MMSE	27.93 ± 1.39	27.53± 1.95	NS
WLM	18.17 ± 3.09	16.76± 4.17	NS
CP	10.38 ± 1.05	9.80 ± 1.90	NS
WLR	5.93 ± 1.41	5.47 ± 1.47	NS
WLRc	9.24 ± 1.02	8.80 ± 1.47	NS
CR	6.86 ± 2.63	6.64 ± 3.00	NS
CERAD total score	70.79 ± 9.45	67.14 ± 11.77	NS

BNT: 15-Item Boston Naming Test; CERAD-K: The Korean Version of Consortium to Establish A Registry For Alzheimer’s Disease; CDR: Clinical Dementia Rating; CP: Constructional Praxis; CR: Constructional Recall; MMSE: Mini Mental Status Examination; NS: Not Significant (for all *p* > 0.2), SD: Standard Deviation; VF: Verbal Fluency; WLRc: Word List Recognition; WLM: Word List Memory; and WLR, Word List Recall.

**Table 2:** Results of voxel-wise functional connectivity analysis.

Region	L/R	Cluster	T Score	*p*-Value *	MNI (x, y, z)		
Group Differences							
Anterior DMN: A-PET-positive group < A-PET-negative group							
Anterior cingulate and middle cingulate cortex	B	867	−3.14	<0.05	6	6	28
Posterior DMN: A-PET-positive group > A-PET-negative group							
Superior parietal cortex and precuneus	R	671	2.96	<0.05	30	−52	56
CEN: A-PET-positive group > A-PET-negative group							
Middle frontal gyrus	R	558	2.70	<0.05	52	00	50
Mean SUVR—functional connectivity relationship							
DMN with Global SUVR							
Anterior DMN: Subgenual anterior cingulate with global SUVR showed negative correlation	B	242	−3.36	<0.05	−4	20	−10
Posterior DMN: Posterior cingulate cortex with global SUVR showed negative correlation	B	377	−4.12	<0.05	8	−34	28
Posterior DMN: Superior parietal cortex and precuneus with global SUVR showed positive correlation	R	269	2.96	<0.05	26	−66	50
DMN with regional SUVR of posterior cingulate cortex							
Posterior DMN: Subgenual anterior cingulate with regional SUVR of posterior cingulate cortex showed negative correlation	B	679	−3.72	<0.0001	8	32	8
CEN with global SUVR							
Precentral gyrus and middle frontal gyrus with global SUVR showed positive correlation	R	393	3.37	<0.01	14	32	4
Graph theory analysis							
Betweenness centrality: A-PET-negative group > A-PET-positive group							
Middle frontal gyrus	L	NA	4.75	<0.05	−38	18	42
Fronto-parietal regions	L	NA	3.71	<0.05	−46	−58	49

*: false discovery rate corrected; B: Both; CEN: Central Executive Network DMN: Default Mode Network; NA: Not applicable; and SUVR: Standardized Value Uptake Ratio.

## Data Availability

The datasets generated for this study are available on request to the corresponding author.
